# Landslide susceptibility mapping using GIS-based statistical models and Remote sensing data in tropical environment

**DOI:** 10.1038/srep09899

**Published:** 2015-04-22

**Authors:** Himan Shahabi, Mazlan Hashim

**Affiliations:** 1 Geoscience and Digital Earth Centre (Geo-DEC), Research Institute for Sustainability and Environment (RISE) Universiti Teknologi Malaysia, 81310 UTM Johor Bahru, Malaysia

## Abstract

This research presents the results of the GIS-based statistical models for generation of landslide susceptibility mapping using geographic information system (GIS) and remote-sensing data for Cameron Highlands area in Malaysia. Ten factors including slope, aspect, soil, lithology, NDVI, land cover, distance to drainage, precipitation, distance to fault, and distance to road were extracted from SAR data, SPOT 5 and WorldView-1 images. The relationships between the detected landslide locations and these ten related factors were identified by using GIS-based statistical models including analytical hierarchy process (AHP), weighted linear combination (WLC) and spatial multi-criteria evaluation (SMCE) models. The landslide inventory map which has a total of 92 landslide locations was created based on numerous resources such as digital aerial photographs, AIRSAR data, WorldView-1 images, and field surveys. Then, 80% of the landslide inventory was used for training the statistical models and the remaining 20% was used for validation purpose. The validation results using the Relative landslide density index (R-index) and Receiver operating characteristic (ROC) demonstrated that the SMCE model (accuracy is 96%) is better in prediction than AHP (accuracy is 91%) and WLC (accuracy is 89%) models. These landslide susceptibility maps would be useful for hazard mitigation purpose and regional planning.

Destructive outcomes of landslides in relation to human life and the overall economic system of many nations around the globe are very severe^1^. Landslide hazard assessment and risk reduction can be accomplished by providing risk managers with easily accessible, continuous, and accurate information about landslide occurrence^2^. Thus, an accurate susceptibility mapping can be key information for a large variety of users from both private and public sectors, from governmental departments and the scientific community on both local and international levels^3^.

Recently, landslide susceptibility mapping has been made possible due to the accessibility and variety of remote sensing data and thematic layers as causative factors data using GIS^4^,^5^,^6^. Most of these landslides are referred to as significant geomorphic processes which usually form an important landscaping aspect in humid tropical mountain surroundings^7^. Records have shown that in Southeast Asia, steep hill slopes, seasonally dry periods, excessive rainfall intensities, and unstable soils are the main causes of frequent landslides^8^. In Cameron highlands, landslide mapping is difficult since the landslides are generally covered by dense vegetation. In addition the cloudy and rainy weather conditions are often undesirable for optical remote sensing data^9^. Consequently, it is necessary that new techniques and accurate data are used in landslide susceptibility mapping in the tropical environment.

In recent years, radar has added a new dimension to disaster management research by providing real-time and precise information. Synthetic aperture radar (SAR) is an active remote sensing system which has the capability of data collection day and night under all weather conditions^10^. In recent years, SAR data are increasingly applied to natural hazards’ researches, either by themselves or in combination with data from other remote sensing sensors^11^. SAR data such as amplitude and SAR polarimetric (POLSAR) images, interferometric DEM and other products of the interferometric DEM have proven useful. The combination of both optical and SAR data can also be utilized in geo-hazards identification and mapping to complement analysis. The SAR technique is highly popular in landslide studies^12^.

Thus, remote sensing can play a role in the production of a landslide inventory map and in the generation of thematic maps related to landslide occurrences. Several previous works have showed the potential of remote-sensing data both in the extraction of causal factors which are linked to landslide occurrences and finding of landslide areas^13^,^14^. Also, reports associated with landslide studies utilizing GIS and probabilistic models have been published^15^. The majority of the previously mentioned research has already been performed while using landslide inventory map extracted from optical satellite images and aerial photographs. Basic qualitative procedures simply use landslide index to identify areas along with related geological as well as geomorphologic features which can be susceptible to landslides^16^.

The usage of the analytical hierarchy process (AHP) approach, produced by Saaty^17^, has been utilized by many researchers world-wide^18^,^19^,^20^. Other different methods have been proposed by several investigators, including weighted linear combination (WLC)^21^,^22^. In addition, spatial multi-criteria evaluation (SMCE) method has also been applied to landslide susceptibility mapping^23^,^24^. Lately, new approaches have been employed for landslide susceptibility mapping including neuro-fuzzy^25^,^26^,^27^, decision-tree (DT) methods^28^,^29^,^30^, and support vector machine (SVM) are tried and their efficiencies are evaluated^31^,^32^,^33^.

Each of the applied approaches depends on different logical explanations with the purpose of generating a landslide susceptibility mapping in an objective manner, thus decreasing the subjective evaluation of the experts. Thus, the primary distinction between earlier studies and the present research is the integration of landslide susceptibility maps extracted by AHP, WLC and SMCE methods for prepared landslide susceptibility mapping in Cameron Highlands area, Malaysia using GIS and remote sensing data. Also, the primary objective of this study is to assess the potential of AIRSAR data together with C-, L- and P-band images and WorldView-1 images for producing landslide inventory maps in the tropical forest namely in the Cameron Highlands, Malaysia.

## Study area characteristics

**Figure 1:**
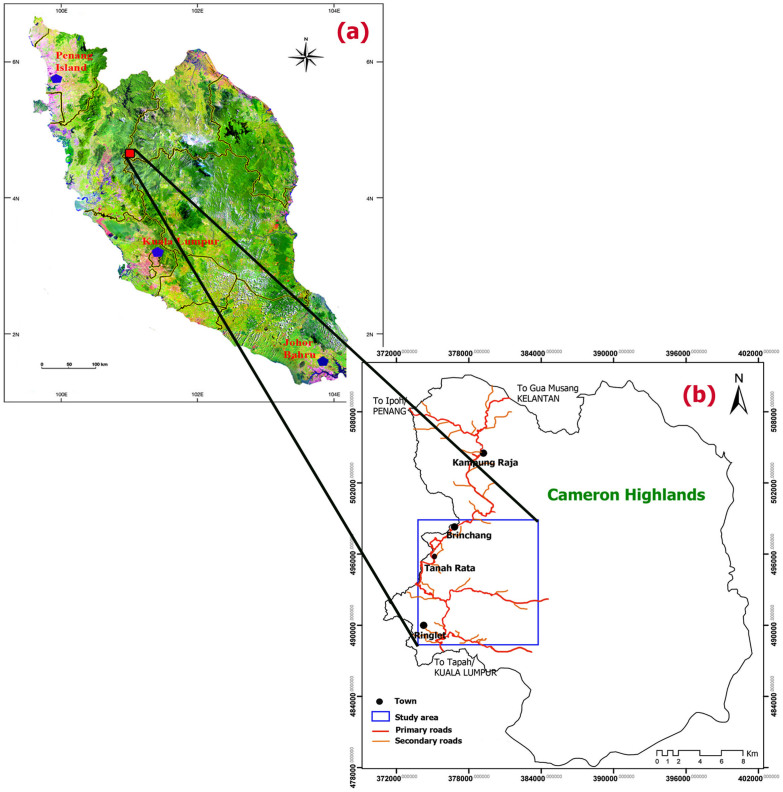
Location of the study area in the Cameron Highlands, Peninsular Malaysia; (a) Landsat ETM^+^ mosaic image of Peninsular Malaysia extracted from ENVI 4.8 software created by first author (H.SH), (b) The rectangular area represents the actual study area in the Cameron Highlands created by first author (H.SH).

With an area of about 660 km^2^, the Cameron Highlands is situated on an undulating plateau within the central section of the Peninsular Malaysia. The research site which is part of Cameron Highlands is bounded by Longitudes 101° 20′ 21″ E to 101° 26′ 50″ E and Latitudes 4° 24′ 37″ N to 4° 33′ 19″ N (Geographic Lat/Lon WGS 84 Projection)^34^. The research area encompasses an area of 38.4 km^2^ and is situated near the northern central part of Peninsular Malaysia in Pahang state which is one of the 13 states of Malaysia (Figure 1).

The geomorphology of the area is rough and has altitude ranging from 840 m to 2110 m. It has an estimated 15% of flat terrain situated adjacent and elongated to the main river. The hilly areas dominate the western and north western parts, of which Mt. Irau is the highest peak with 2110 m. Bertam and Telom Rivers are the main drainage features in this area. Its valley and tributaries are mainly flowing from north-northwest to south-southeast^35^. Megacrystic biotite granites are the most common geological structure of central mountain chain in Peninsular Malaysia^36^. Schists, phyllite, slate, as well as limestone are also significant lithologies of Cameron Highlands^37^. The highlands are usually cloud-covered during the year. The tropical forest and tea plantations, temperate vegetable and flower farms region are the major vegetable cover in the study area^38^.

The average annual rainfall of between 2,500 and 3,000 mm per year in the Cameron Highlands was calculated between March and May and from November to December. The volume of rain is another agent which impacts the fill slopes, leading to stream and gully erosion^38^. Average daytime and nighttime temperatures in the study area of 24°C and 14°C respectively, locates the Cameron Highlands in moderate climatology category. Physically, 66% of the slope gradients are more than 20°. About 8% (5,500 ha) are categorized under agriculture, 86% (60,000 ha) of the area is forested, 4% (2,750 ha) is occupied by housing, and the residuum are used for recreation and other activities. The impact of present agricultural activities has led to severe soil erosion, culminating into large quantities of sediments. In addition, occurrences of landslides are another hazardous natural disaster that threatens this location which has high economic potential^39^.

In the Cameron Highlands, Malaysia, like in most tropical mountainous areas, natural hazards such as flash floods, mass movement and landslides are of great social concern and cause considerable damage to life and property. Earthflows, mudflows and landslides present a major danger in Cameron Highlands because of the nature of the topography, the climate and human activities. Generally, precipitation is a triggering factor that can cause mudflows and landslides^38^. Landslides in Cameron Highlands have also destroyed the roads and troubled economic activity such that total economic loss due to landslides was approximately US $1 billion between 1973 and 2007^39^.

## Methods

### Landslide inventory

Landslide inventory maps show locations and also features of landslides that have moved in the past although usually do not show the mechanism(s) that triggered them. Therefore, inventory maps provide useful information about the spatial distribution of locations of existing landslides and the potential for future landslides^40^. Landslide mapping is difficult in tropical mountainous environments as dense vegetation growth obscures landslides soon after they occur^9^.

Sometimes carrying out mitigation measures is held back due to insufficient and dependable landslide inventory map which hampers the evaluation of landslide hazard and risk. Utilizing remote-sensing data such as radar, optical satellite images and aerial photography interpretation are primary methods to obtain important, cost-effective information of landslide location^14^. The landslide information taken from remotely sensed images is especially associated with morphology, plant life, and hydrologic conditions of the region^6^.

Several types of remote sensing data may be used in detecting landslide features, for instance, stereo-remote sensing products which in turn reveal the actual morphodynamical features of landslides^41^. In this study, published reports, field surveys, interpretation of digital aerial photographs (DAP) (10,000–1:50,000 scale) over a 25-year period, WorldView1 satellite imagery on the March 2011, AIRSAR data on November 2004 has been utilized for extraction of landslide inventory map.

**Figure 2:**
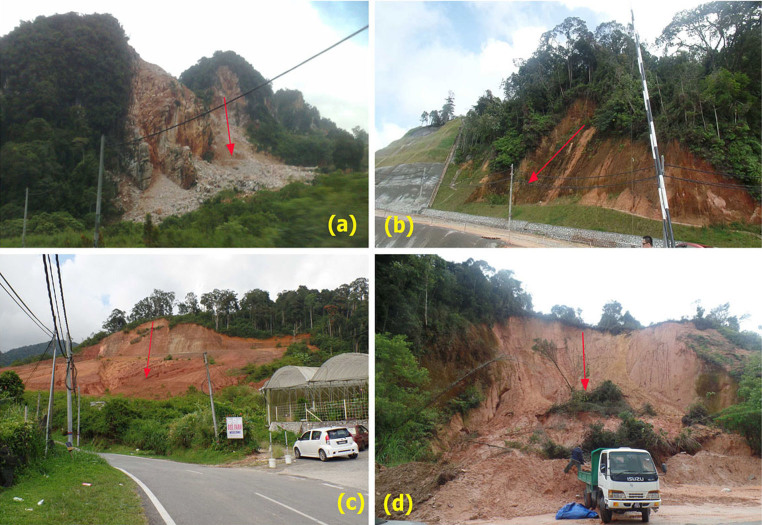
Field photographs of recently occurred landslides and types of landslides that were taken from field surveys by first author (H. SH); (a) a shallow translational rockslide, (**b**) a shallow translational debris slide at the road side, (**c**) and (**d**) deep-seated rockslides. Arrow depicts the movement direction.

These black and white digital images with resolution = 0.54 m pixel were taken during 1981–2006 and were acquired from the Malaysian Surveying and Mapping Department archives. WorldView-1 satellite data, which has a resolution of 0.46 meter for panchromatic band was used for detection of occurred landslides and validation of landslide inventory map obtained from AIRSAR data in the study area. The AIRSAR data were collected over the study area in November 2004, during the PacRim1 campaign. This dataset is to be compared with landslide features generated from aerial photographs and WorldView-1 satellite imagery which are also generated in UTM reference system. Multiple field investigations and ground control points (GCPs) were carried out by using global positioning system (GPS) for collection of mapping information on landslide locations (See Figure 2) and generating stereo models from digital aerial photography data.

To identify landslides in the study area, three techniques were employed. The first technique was to compare directly by overlaying landslide vector images onto the DEMs and AIRSAR raster images. The second technique was to classify the images using ENVI 4.8 software. This is to separate landslides from the other land cover types in the surrounding area. The last technique is to separate landslides from the other land cover types in the surrounding area using segmentation followed by classification. This technique was carried out using software called “eCognition”. In eCognition software pixels are segmented into image object, so the image classification process in this software is image objects based rather than pixels based classification that was previously carried out in ENVI 4.8 software^42^.

The data used in segmentation were 1) C-, L- and P-band (wavelengths) with seven polarizations (Chh, Lhh, Lvv, Lhv, Phh, Pvv and Phv) images and 2) Slope image. In segmentation the information about the group of pixels inside the boundaries of landslide was used. The information include: 1) Spectral values of the C-, L- and P-band and 2) Average slope of the area. These spectral values may represent spectral signatures of landslides. In segmentation processes using eCognition software, all three bands with seven polarizations and a slope image were combined to identify landslides in the images. If one existing landslide can be identified it can be used in the segmented image as a sample polygon. Using classification techniques, all other polygons that have the same characteristic of pixels brightness and average slope will be highlighted as similar^42^.

Furthermore, the efficiency and quality of the SAR data and optical satellite images should be examined using a proper method. The root mean square error (RMSE) method was used for accuracy of the obtained result^43^,^44^. The each ground control points, the efficiency of the SAR data and optical satellite images is calculated based on the formula^43^:
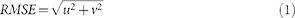
where *u* is residual in the *x* axis; *v* is residual in the *y* axis.

Total RMSE is then derived as (Eq. 2):


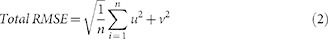
 where *n* is number of GCPs; *u* is residual in the *x* axis; *v* is residual in the *y* axis.

The total RMSE was calculated for each area, SAR and optical satellite images based on nearest neighbour resampling method^45^.

### Spatial database construction

Data collection is the main step in landslide susceptibility mapping whereby the relevant landslide conditioning factors are extracted to construct a spatial database. These processes are subsequently evaluated by using the relationship between the landslide and landslide causative factors, and then verification of the results^25^. There are no universal guidelines regarding the selection of factors in landslide susceptibility mapping. One parameter may be an important controlling factor for landslide occurrence in a certain area but not in another one. The selection of causal factors therefore needs to take the nature of the study area and data availability into account. Collectively ten parameters of slope, aspect, soil, lithology, NDVI, land cover, distance to road, distance to drainage, precipitation, and distance to fault were used to construct a spatial database using GIS, SAR data and optical satellite images processing. The database consists of vector-type spatial datasets derived from Arc GIS 9.3 software package.

In the first step a digital elevation model (DEM) of the research area was produced from the triangulated irregular network (TIN) model using AIRSAR DEM with 10-m pixel size. The slope and slope aspect parameters were obtained from the generated AIRSAR DEM with 10-m pixel size. In addition, the distance from drainage was calculated using the AIRSAR DEM. The fault lines and lithology were derived from geological map with 1:63,300-scale from Mineral and Geosciences Department of Malaysia. Also, the lineaments were derived from the structural maps and the aerial photos. The distance from the road was calculated using the 1:25,000-scale topography map. A 50-m buffer zone is chosen as a distance from road in the study area that is determined based on the occurred landslides to the closeness of the road. The soil types were acquired from a 1:25,000-scale soil map. In this research, land cover was extracted from SPOT 5 satellite image on 21 March 2010 that was calibrated by using ground control points (GCPs) obtained during field works. The supervision classification method by using ENVI 4.8 software was used to develop a statistical characterization of the reflectance for each information class of land cover map.

Furthermore, the SPOT 5 data were first classified into eight main land cover types using a supervised maximum-likelihood classification (MLC), namely grass, primary forest, rubber, cutting, secondary forest, settlements, agricultural area, and water body. Field surveying was also used to justify the land cover map’s accuracy according to the order of SPOT 5 spatial resolution (~ 10 m).

Despite image pre-processing (geo-referencing and ortho-rectification, geo-rectification) on the SPOT 5 images, a 21 ground control points (GCPs) obtained during field visits were used for additional improvement in the accuracy of satellite images. Also, the SPOT 5 satellite image was used for extraction of normalized difference vegetation index (NDVI) map. The NDVI value was calculated using the formula NDVI = (IR − R)/(IR + R), where IR and Red are the near infrared (NIR) and red bands, which are from 0.7 to 1 lm and 0.6 to 0.7 lm of the electromagnetic spectrum. The NDVI value, which indicates the presence and intensity of vegetation in the study area, was classified into ten classes.

**Figure 3:**
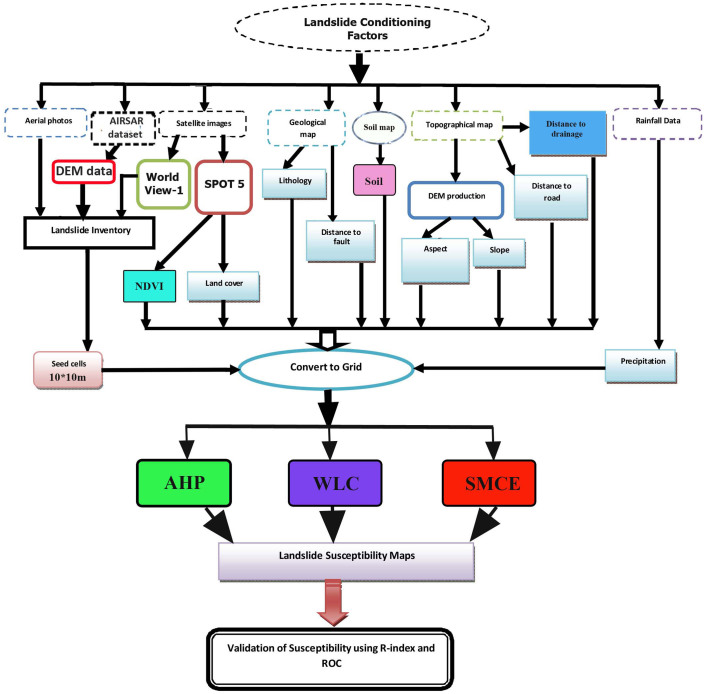
Different steps of preparing the map for landslide susceptibility mapping.

The precipitation data was prepared using the last 30 years (1981–2011) of historical rainfall data. In our methodology we used long-term precipitation for a 30 year period. An average annual rainfall contour map is mapped out from the daily rainfall data measurements. Also, the Kriging method using Arc GIS 9.3 was used for spatial interpolation on the contour maps. The flowchart for the landslide susceptibility mapping and spatial data are shown in Figure 3. All the landslide related factors were converted to a raster grid (10 × 10-m cells) that included 1,725 rows by 5,621 columns for application of the three different GIS-based statistical approaches including analytical hierarchy process (AHP), weighted linear combination (WLC) and spatial multi-criteria evaluation (SMCE).

### Landslide susceptibility mapping models

Landslide susceptibility analysis was implemented using the analytical hierarchy process (AHP), weighted linear combination (WLC) and spatial multi-criteria evaluation (SMCE) methods in a part of the Cameron Highlands, Malaysia using GIS- based statistical models and remote sensing data. Also, the maps credibility was validated using R-Index and ROC methods.

### Analytical hierarchy process (AHP) in landslide susceptibility analyses

The AHP developed by Saaty^17^ is a flexible tool of analyzing complicated problems focusing on site selection, urban planning, and landslide susceptibility analysis^21^. AHP which is a multi-criteria decision-making and multi-objective approach allows the active participation of decision-makers namely the managers in reaching an agreement rationally^46^.

These factors are arranged in a hierarchic order and numerical values to subjective judgments based on the relative importance of each factor. Subsequently these factors are synthesized and each factor is assigned according to their importance^47^. Apart from that, reciprocal pair-wise comparison matrix is established to utilize AHP. Each layer based on a 9-point rating scale are the entries into the matrix as developed by Saaty^48^ (See Table 1).

Generally, the specification of the values of the factors relative to each other is affiliated to the selection of the decision-maker. Nonetheless, in this research, both the determination of the decision options and comparison of the parameters were based on the comparison of landslide inventory maps^18^. The weight of each factor from the matrix weighting factor was multiplied by its weight class. The result of the susceptibility map is ascertained by factors with high local representation. These representations can be based on different parameters including natural (lithology, distance to faults, etc.), man-made (roads and other engineering structures), causal (slope, aspect, lithology, etc.) and triggering (precipitation, seismicity, etc.)^18^,^19^,^20^,^21^,^22^,^23^,^24^,^25^,^26^,^27^,^28^,^29^,^30^,^31^,^32^,^33^,^34^,^35^,^36^,^37^,^38^,^39^,^40^,^41^,^42^,^43^,^44^,^45^,^46^,^47^,^48^,^49^. The selection of the ten causal factors in this study is based on these mentioned four criteria, and also data accessibility. In AHP approach, consistency ratio (CR) Eq. (3), is utilized to show the probability that the judgments matrix was randomly created^48^.


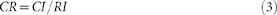
where *RI* is the average of the resulting consistency index depending on the order of the matrix given by Saaty^48^ and *CI* is the consistency index and can be expressed as Eq. (4)
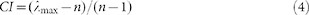
where *λ_max_* is the largest or principal eigen value of the matrix and can be easily calculated from the matrix and *n* is the order of the matrix.

If the CR values were greater than 0.1, the AHP model was automatically rejected. The acquisitive weights were employed by using a weighted linear sum procedure. Furthermore, the acquisitive weights were employed to calculate the landslide susceptibility models^50^.

### Weighted linear combination (WLC) in landslide susceptibility analysis

Weighted linear combination (WLC) is a hybrid between qualitative and quantitative methods^21^. WLC is based on the qualitative map combination approach (heuristic analysis). This technique is a popular method that is customized in many GIS and is applicable for the flexible combination of maps. Thus the tables of scores and the map weights can be adjusted based on the expert’s judgement in the domain. First, this method requires the standardization of the classes in each factor to a common numeric range. Each factor rating was based on the relative importance of each class according to field observations and existing literature, indicating the conditions as highly susceptible to slope failure^51^.

Primary-level weights and secondary-level weights are two types of parameters weights used^15^. The primary-level weights are rule-based whereby the ratings given to each class of a parameter is based on certain criteria. In this research, this criterion can be described as landslide density. In WLC method, the landslide density is a ratio between the area of landslide pixels situated inside a category of a specific factor divided by the total area of that category. The obtained result of this criterion is converted into percentage. The secondary-level (factor weights) determine the degree of exchange of one parameter versus another parameter based on opinion-based scores^21^. Both the parameters weights are combined to estimate landslide susceptibility and classify areas in relative susceptibility categories^21^,^22^,^23^,^24^,^25^,^26^,^27^,^28^,^29^,^30^,^31^,^32^,^33^,^34^,^35^,^36^,^37^,^38^,^39^,^40^,^41^,^42^,^43^,^44^,^45^,^46^,^47^,^48^,^49^,^50^,^51^,^52^.

Susceptibility *S* (*i, j*) in each pixel (*i, j*) can be expressed as the combination of the product of primary and secondary level weights Eqs. (5 and 6)^52^:
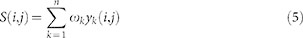


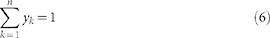
where 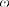 is the primary-level weight of parameter *k*, and *y* is the secondary-level weight of parameter *k*.

The weights of ‘proportional importance’ to each attribute map layer are directly affected by the decision-maker. A total score is then obtained for each alternative. This is done by multiplying the weight allocated to each attribute by the scaled value and summing the outputs of all attributes. In this method, highest overall score can be selected from the overall scores calculated for all of the alternatives^53^. The final steps for creating the landslide susceptibility map using WLC method is the combination of all weighted layers into individual maps. Then, landslide susceptibility zones were generated based on classification of the scores of these maps^21^. The WLC method can be performed using any GIS system that has overlay techniques.

### Spatial multi-criteria evaluation (SMCE) in landslide susceptibility analysis

Spatial multi-criteria evaluation (SMCE) application helps and allows users to perform multi-criteria assessment in a spatial approach. In SMCE, the alternatives are locations in the form of points, lines, areas, and grid cells. Therefore, criteria could occur in the form of maps^54^. Thus, SMCE is an applied science-based method that combines spatial analysis using GIS and multi-criteria evaluation (MCE) to transform spatial and non-spatial input which generates output decision^55^. Spatial multi criteria evaluation is considered as a procedure that includes the input layers, which are the spatial representation of the criteria. They are a ‘criteria tree’ that can be standardised, grouped and weighted. In addition, the input layers (resultant decision), need to be standardised from their original values to the value range of 0–1. Furthermore, the indicators have cartographic representations (natural and administrative polygons and pixel based raster maps) and different measurement scales (nominal, ordinal, interval and ratio)^23^.

The output of SMCE is one or more ‘composite index map(s), which indicates the extent to which criteria are met or not in different areas, and thereby supports decision making^56^. The multi-criteria evaluation of AHP method has been used as the theoretical background of SMCE method. There are several phases in conducting the SMCE, such as problem tree analysis, standardization, weighting, and map generation. Once all the criteria and related maps or attribute tables are entered in the criteria tree, the criteria have to be standardized. The values in the various input maps have different meanings, and are probably showed in different units of measurement such as percentages, meters, distance in meters, land cover classes, etc.^24^. In order to standardize input maps in SMCE environment, one of the standardization methods such as numerical, Boolean, and qualitative methods can be used.

The first step for standardizing map values is to convert the actual map values to a range between 0 and 1 by using a set of equations. The next step is the determination of each indicator for intermediate or overall objectives. Finally, the landslide conditioning factors are weighted by means of direct, pairwise, and rank ordering comparison, and the output is a composite index map^23^.

## Results and Discussion

### Landslide location detection using AIRSAR data and optical satellite images

The size and locations of landslides on the old landslide map in the study area are inaccurate, therefore not suitable for any applications in the landslide inventory map. Also, identified landslide is too small. Therefore the information about the pixel values might be insufficient for identifying landslide by segmentation and classification technique. Landslides can be identified on AIRSAR intensity image. The spectral values of the pixels representing landslides can be differentiated from the spectral values of the surrounding.

**Figure 4:**
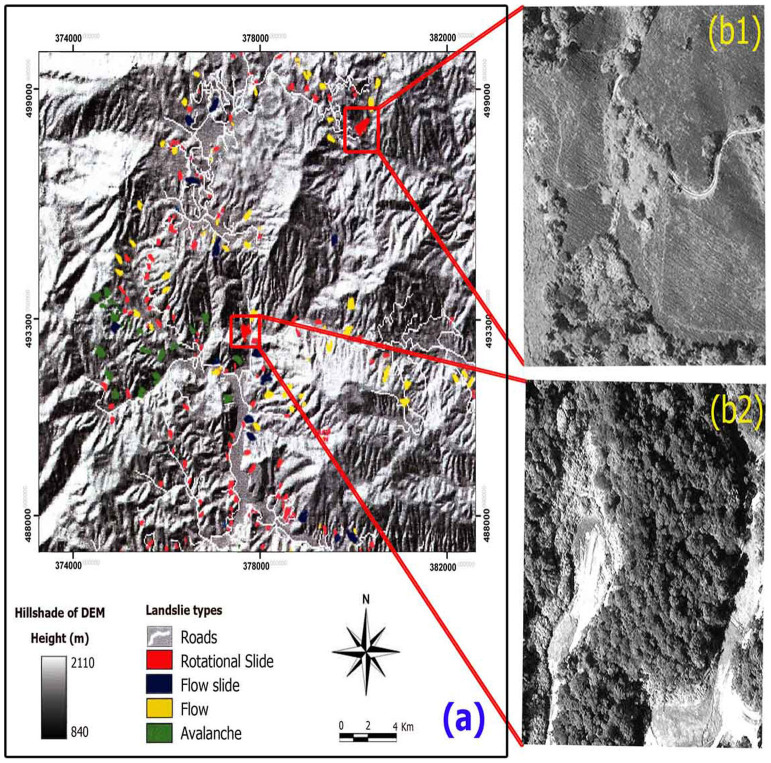
Final landslide inventory map extracted from AIRSAR data and WorldView-1 satellite images; (a) types of landslide locations overlaid on AIRSAR DEM, (**b**) enlarged views of two landslides locations on WorldView-1 satellite images (panchromatic band).

Because of many problems with the old landslide map, validation of the map was the only way to check the accuracy. The landslides that were identified on the ground as mentioned previously were also identified on aerial photograph. To validate the landslide inventory map, the landslide features that were obtained from WorldView-1 satellite images were overlaid onto the C-, L- and P-band image. Since the landslide feature was in UTM reference system, C-, L- and P-band image in the UTM reference system was used. The final landslide inventory map that was compiled in the present study is shown in Figure 4.

Subsequently, the final landslide vector map was transformed into a grid database with a cell size of 10 × 10 m in ArcGIS 9.3. The compilation of more than 25 years of landslide inventory resulted in a total of 92 landslides identified, covering an area of 6.27 km^2^, and accounting for 4.05% of the study area. The minimum, mean and the maximum landslide areas are 0.003, 0.017 and 0.123 km^2^ respectively. Among the cases of landslide occurrences, 74 cases (80%) were selected during the training of landslide susceptibility mapping models and the remaining 18 cases (20%) were used for validation purpose.

The results also identified that most of the landslides detected from AIRSAR data, digital aerial photographs and WorldView-1 satellite images are shallow rotational, and there are a few translational and flow types. In the present study, only the rotational landslides are counted, because the occurrences of the other types of landslides were scarce and very small. To assess the correctness and quality of the result, root mean square errors (RMSE) (Eqs. 1 and 2) was performed by comparing the in-situ measurements with the resulted one. The resulted RMSE was 0.163 which is acceptable and shows the efficiency of the proposed method in recognizing the landslides with high precision.

### Landslide susceptibility mappings using GIS-based statistical models

The ten factors were transmuted into a vector-type spatial database using the Arc GIS 9.3. Also, the landslide triggering factors were extracted from the database. Furthermore, in this study, the landslide susceptibility analyses were performed using the GIS-based statistical models including AHP, WLC and SMCE.

### Landslide susceptibility mapping using AHP model

The rating values for each class of each triggering factor which had an influence on landslide susceptibility were also calculated by Expert choice software. The software calculates the weight of each factor and based on this weighting factors were prioritized. To get factor weights in AHP, the same method used in calculating the rating value was applied as shown in Table 2. The software also calculates the CR. The ratio implies an acceptable level of consistency in the pair-wise comparison which is sufficient to recognize the factor weights in the landslide susceptibility model. In this research the CR value was less than 0.1 for all cases of the acquired class weights. This means a reasonable level of consistency in the pair-wise comparison.

Precipitation with AHP weight (0.142), Lithology with AHP weight (0.124), and slope with AHP weight (0.109) were found to be important parameters in occurrences of landslides in the study area, whereas distance to fault (0.074) and distance to road (0.078) received a low degree of importance. The results of the class weights, factor weights and consistency ratio of the data layers are shown in Table 2.

As a result of the AHP analysis, we used the following equation;


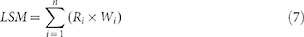
 where *R_i_* is the rating class of each layer and *W_i_* is the weights for each of the landslide conditioning factors.

**Figure 5:**
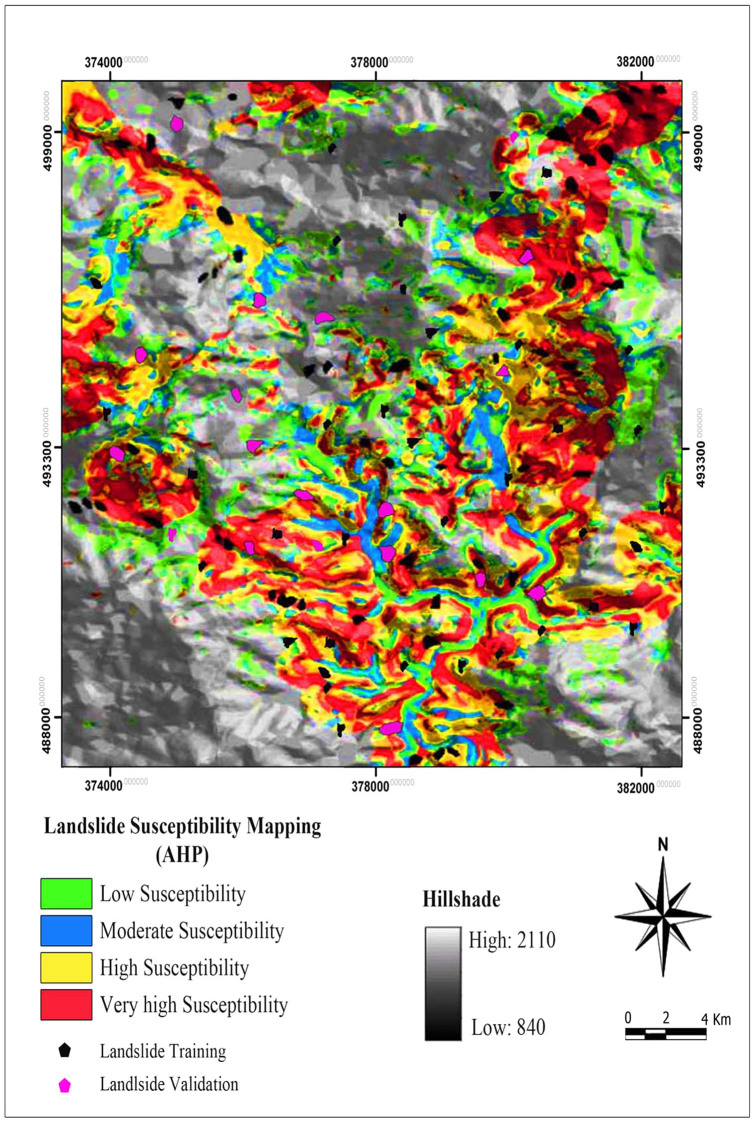
Landslide susceptibility map derived from AHP model.

These LSM values were then divided into four susceptibility zones including low (LS), moderate (MS), high (HS), very high (VHS) based on the natural breaks method (See Figure 5).

Based on the result of the obtained landslide susceptibility map, 28.32% (1.77 km^2^) of the total area show low landslide susceptibility. Moderate, high and very high susceptible zones make up 36.23% (2.27 km^2^), 24.18% (1.70 km^2^) and 11.27% (0.70 km^2^) of the total area, respectively (See Figure 5).

### Landslide susceptibility mapping using WLC model

After producing the landslide related parameter maps and the weights for their classes, this section describes the combination of the maps to estimate landslide susceptibility in the target area using the WLC approach. To be able to create a landslide susceptibility map using WLC model, the factor weights were first extracted from AHP method that are principally based on the ratings provided to each class of a factor. In order to actualise this phase, the pair-wise comparison matrix and CR of used data layers are shown in Table 3.

In this study, the weight value of the precipitation (0.141) and slope (0.123) are the highest. On the other hand, the low WLC weights belong to distance to fault and NDVI with 0.062 and 0.073, respectively (See Table 3). The CR is ascertained to be 0.069 and this value expresses the appropriate amount of CR employed to acquire the comparison matrix because it is less than 0.1. Therefore the weights related to factors were multiplied by the appropriate factor maps and then all the weighted factor maps were overlaid to extract a landslide susceptibility map based on WLC model.

**Figure 6:**
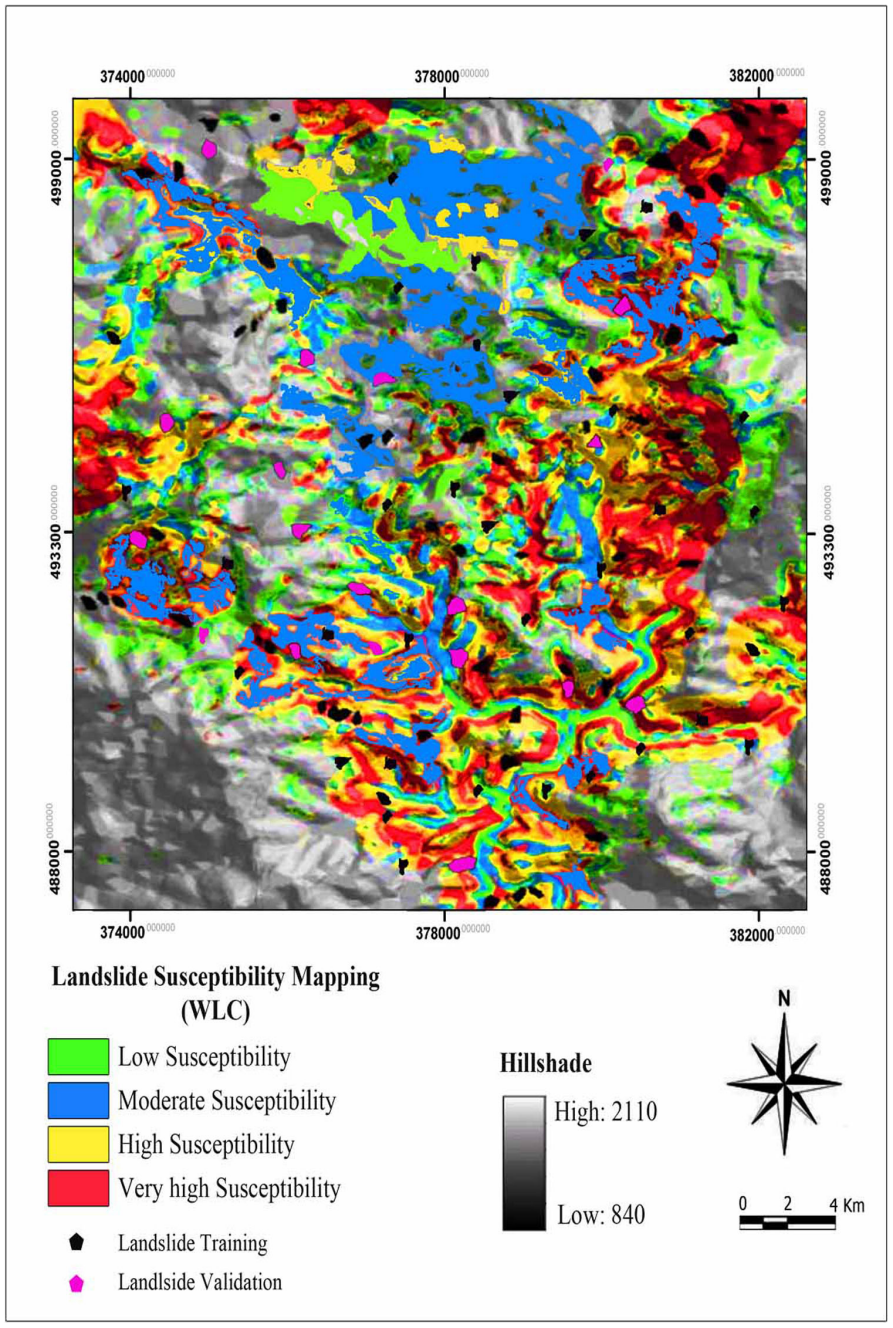
Landslide susceptibility map derived from WLC model.

The allocated rates used to reclassify vector data layers and raster data layers were generated from new reclassified data. Also, the raster calculator function was used to reclassify raster layers as input parameters. By means of the raster calculation all the weighted factor maps were added to provide the final landslide susceptibility map. Based on this method, four susceptibility zones were identified such as low, moderate, high and very high (See Figure 6).

The WLC-derived landslide susceptibility map yields very high susceptible zones of about 7.10% (0.44 km^2^) of the total area while about 17.12% (1.07 km^2^) is classified as being highly and moderately susceptible and 46.56% of the case study area (2.91 km^2^) is classified as being a moderate susceptible zone. 22.34% of the study area (1.40 km^2^) is classified as low susceptible zone. Total susceptibility value in each cell was the sum of the rasters (corresponding to primary weights) multiplied by their secondary weights, as expressed in Eq. 5. The categorized susceptibility map was compared to the landslide inventory map to calculate the area of landslides in each class (See Figure 6).

### Landslide susceptibility mapping using SMCE model

The first stage to generate SMCE analysis is standardization of input layers based on their original values (0–1). This standardization can be performed as a SMCE module in ILWIS (Integrated Land and Water Information System) software to acquire the composite index maps and the final landslide susceptibility map^57^.

The SMCE was built based on analyzing the weight value in bivariate statistical analysis for classes of conditioning factors (See Table 4). In this research, for standardization of the scale in thematic layers the fuzzy logic method was used. All comparisons are based on analytical hierarchy process (See Table 1). The employment of fuzzy sets to represent linguistic terms makes it possible for one to represent more effectively and continuously something which is fuzzy. In this research, in order to input parameters of SMCE in linguistic form, the following fuzzy sets were utilized:

- Low = (0/1, 0.25/2, 0.75/3, 1/4)
- Moderate = (0/1, 0.5/2, 1/3, 0.5/4)
- High = (0/1, 1/2, 0.75/3, 0.25/4)
- Very high = (0/1, 0.25/2, 0.5/3, 0.75/4)

In the foregoing fuzzy set notation, the values before the slash are the degrees of confidence and the values after slash are the members of the set^24^. The fuzzy set values of the predisposing factors of the landslides for study area are found as follows:

- μ_S_ Slope = (0.09/1, 0.73/2, 0.82/3, 0.91/4, 1/5)
- μ_S_ Aspect = (0/1, 0.87/2, 1/3, 0.75/4, 0.96/5, 0.39/6, 0.46/7, 0.65/8, 0.54/9)
- μ_S_ Soil = (1/1, 0.81/2)
- μ_S_ Lithology = (1/1, 0.87/2)
- μ_S_ NDVI = (1/1, 0.93/2, 0.72/3, 0.83/4, 0.49/5, 0.87/6, 0.42/7, 0.64/8, 0.77/9, 0.57/10)
- μ_S_ Land cover = (0.88/1, 0.56/2, 0.68/3, 1/4, 0.96/5, 0.92/6, 0.79/7, 0.62/8)
- μ_S_ Precipitation = (0.33/1, 0.81/2, 0.48/3, 0.54/4, 0.68/5, 0.76/6, 0.95/7, 0.71/8, 0.88/9, 1/10)
- μ_S_ Distance to road = (1/1, 0.95/2, 0.84/3, 0.79/4, 0.71/5)
- μ_S_ Distance to drainage = (1/1, 0.89/2, 0.94/3, 0.84/4, 0.76/5, 0.71/6, 0.63/7)
- μ_S_ Distance to fault = (1/1, 0.82/2, 0.94/3, 0.79/4, 0.71/5, 0.62/6)

In this research, grid-based analysis was performed in Arc GIS 9.3 to produce the fuzzified index maps. Criteria for landslide susceptibility mapping in this research are divided in four groups (sub-goals) such as geomorphological (slope, aspect); geological (distance to fault and lithology); environmental (soil, land cover, distance to road and NDVI); hydrological (distance to drainage and precipitation) factors. They are the input for the SMCE analysis.

The levels of the influence of sub-goals and weight value of main indicators for the study area were calculated by AHP (See Table 4). Based on our results in expert choice software, it can be seen that hydrological factor has the most influence on landslide occurrence (0.146). On the other hand, the geological factor which has less influence was categorized in the lowest level (0.871). Based on the results in Table 4, in geomorphological factors, it can be seen that slope factor with weight value (0.128) is more susceptible to landslide but the aspect is less prone to landslide as it has weight value of 0.112. For geological factors, weight corresponding to lithology (0.097) is large, whereas distance to fault is lowest (0.073). In the case of environmental factors, it was observed that soil, land cover, distance to road, and NDVI have a weight value of 0.123, 0.084, 0.083 and 0.087, respectively. In hydrological factors, precipitation has a higher probability of occurrence than the distance from drainage and therefore received a higher weight (0.143 vs. 0.081).

**Figure 7:**
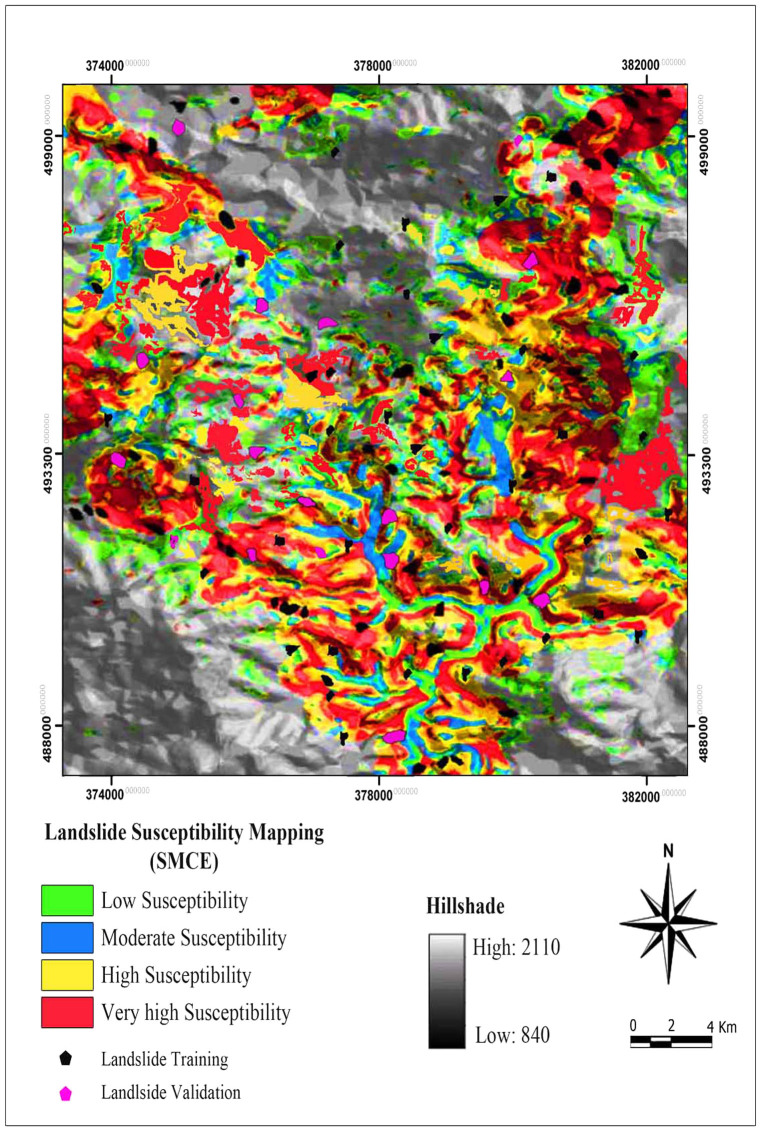
Landslide susceptibility map produced by the SMCE model.

As a general result, the precipitation is highly prone to landslide occurrence, and in contrast, distance to fault has the lowest impact in landslide susceptibility. For all cases of the acquired class weights (sub-goals and indicators), the consistency ratios are less than 0.1; the ratio indicates appropriate degree of consistency that was good adequate to recognize the class weights. The final landslide susceptibility map extracted from SMCE model was reclassified into four relative susceptibility zones: low, moderate, high, and very high (Figure 7) based on natural break classification method.

According to the landslide susceptibility map acquired from the SMCE approach, 10.16% (0.63 km^2^) of the entire area is found to be of low landslide susceptibility zone. Moderate and high susceptible zones showed 13.18% (0.82 km^2^), and 29.14% (1.82 km^2^) of the entire area, respectively. The very high landslide susceptibility zone is 47.52% (2.97 km^2^) of the entire study area (See Figure 7).

### Evaluation, comparison and precision of landslide susceptibility mapping methods

In this research landslide inventory map is used for methods evaluation and comparison of susceptibility mapping. In order to do that, we crossed the above mentioned map with map of susceptibility in the geographical information system, and landslide index was used for evaluation of hazard classes on zoning maps (Eq. 8). Landslide index is defined as follows^58^.


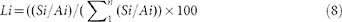
where *Li* is the index for occurrence of landslide hazard in each susceptibility zone (percent), *Si* is the slide area in each susceptibility zone, *Ai* is the area of each zone, and *n* is the number of susceptibility classes.

In the above relation: landslide index is percentage of sliding surface ratio in any zone to the area of that zone, divided by the total sliding ratio to the total surface of the zones. In order to compare the obtained susceptibility maps, the parameter considered for precision of the predicted results is (*P*). Precision of the predicted results can be estimated by the following (Eq. 9)^59^.
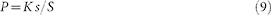
where *Ks* is the area of slide zone in upper moderate susceptibility level and *S* is the area of landslide in the region.

As Table 5 shows, from low up to very high level, *Li* amounts increase in all of the methods used. Therefore, all of the methods used for susceptibility levels have yielded acceptable results. In Table 5, the *P* refers to the high efficiency of SMCE and AHP in susceptibility mapping in the studied region. From the statistical methods that were used, respectively, precision of the method (*P*) were 96% for SMCE, 91% for AHP and 89% for WLC which are all compatible with the conditions for occurrence of landslides in the region.

### Validation of landslide susceptibility maps using R-index and ROC methods

The landslide susceptibility analysis was carrying out using three different approaches such as AHP, WLC and SMCE models. Furthermore, the analysis results were validated using the Relative landslide density index (R-index) and receiver operating characteristic (ROC) analysis to evaluate the correlation between the landslide susceptibility maps and landslide inventory points. Then, among the types of landslide occurrences, 74 cases (80%) had been determined throughout the training of landslide susceptibility mapping models and 18 cases (20%) had been employed for validation purpose. Validation of landslide susceptibility maps is performed with a formula defined by^60^ (Eq. (10) as follows:


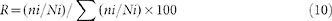
 where *ni* the number of landslides occurred in the sensitivity class *i* and *Ni* the number of pixels in the same sensitivity class *i*.

**Figure 8:**
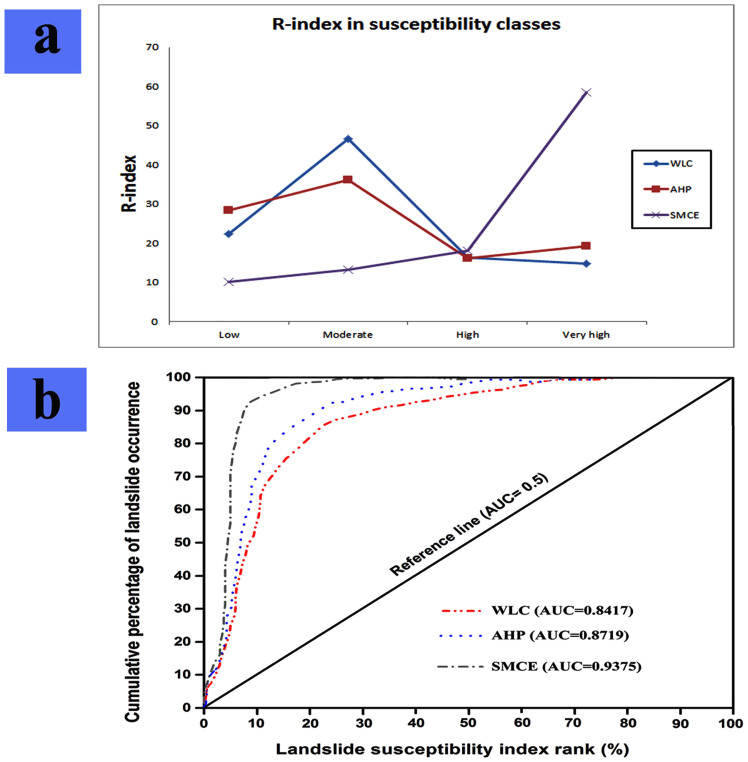
Validation of landslide susceptibility maps, (a) R-index validation of three methods for landslide susceptibility mapping, (b) Prediction accuracy assessment and success rate curve of the constructed landslide susceptibility models.

The results obtained from SMCE and AHP analysis methods have been more coherent with recurrent landslides occurring in sensitivity classes. In the characteristic analysis map, all susceptibility classes are correlated with the distribution of landslide occurrence. From the classes with very-high susceptibility, SMCE and AHP methods were better than WLC method. The R-index sample data sets for classes of very high hazard in AHP, WLC and SMCE maps are 19.31%, 14.75%, and 58.49% respectively. From assessment of all classes, SMCE was more accurate than the other two methods. The results of validation (R-index) of AHP, WLC and SMCE models are shown in Figure 8a.

The suitability of each model was also assessed by comparing the maps with known landslide locations. The area under the receiver operating characteristic (ROC) curve, known as the AUC and ranging from 0.5 to 1.0, is widely employed to estimate the accuracy of presence or absence predictive models^20^. This curve was obtained by means of the statistical analysis software SPSS.

The success rates of the verification for the three LSM methods including AHP, WLC and SMCE are shown in Figure 8b. The results obtained from Figure 8b shows that a value of AUC for AHP was 0.8719 and the prediction accuracy was 87.19%. In the WLC and SMCE methods, the area ratios were 0.8417 and 0.9375 and the prediction accuracy for WLC and SMCE were 84.17% and 93.75%. These results obtained from R-index (Figure 8a) and ROC (Figure 8b) indicate that the SMCE model looks to be more accurate in terms of the performance of landslide susceptibility mapping and has better prediction accuracy than the other two models in the study area.

## Conclusion

Landslide susceptibility mapping in tropical mountainous areas is usually difficult because of inadequate approachability, the vegetation, and cloudy weather situations. In the present research, a landslide susceptibility evaluation was performed in a part of Cameron Highlands, Malaysia. The research involved three main phases including landslide inventory analysis, susceptibility mapping, and validation. The landslide inventory map with a total of 92 landslide locations revealed the strong capability of AIRSAR data and WorldView-1 images satellite images to distinguish and detect the very small landslides of the earth surface which occurred due to the heavy precipitation with resulted accuracy of 0.163 RMSE. This RMCE accuracy is acceptable and shows the efficiency of the proposed method in recognizing the landslides with high precision.

Landslide types are very diverse, and as a consequence landslide susceptibility assessment can only be achieved by specific landslide models. Hence, the determination of dominant landslide types and selection of appropriate suitable landslide models for landslide susceptibility mapping is difficult and will strongly influence the final landslide susceptibility map. In this study, the potential of GIS-based statistical models such as analytical hierarchy process (AHP), weighted linear combination (WLC) and spatial multi-criteria evaluation (SMCE) methods using GIS tools and remote sensing data have been evaluated in the study area. Ten factors as slope, aspect, soil, lithology, NDVI, land cover, precipitation, distance to fault, distance to drainage, and distance to road were obtained from the spatial database. The selected causative factors for the study area were considered carefully based on the relevance, availability and scale attributes. However, some statistical analysis approaches require that the causative factors possess certain properties as conditional independence. Hence, the number of causative factors that can be used as input for landslide susceptibility modeling may be reduced depending upon the conditional circumstances. Therefore, accurate landslide susceptibility mapping on a regional scale strongly depends on the selection of landslide causative factors, which can be quite different according to various researchers.

In the AHP model, the precipitation (weight = 0.142) and lithology (weight = 0.124) parameters are positively associated with the occurrence of landslides. In the WLC model, the weight value of the precipitation (0.141) and slope (0.123) are the highest. On the other hand, the low WLC weights belong to distance to fault and NDVI with 0.062 and 0.073, respectively. Based on obtained results from SMCE model, it can be seen that hydrological group has the most influence on landslide occurrence (0.146) in this group and that precipitation has a high weight (0.143). On the other hand, the geological group which has less influence was categorized in the lowest level (0.871) and in this group, high weight belonged to lithology (0.097). As a general result extracted from SMCE model, precipitation had the highest impact on landslide occurrence, and in contrast, distance to fault had the lowest impact in landslide susceptibility.

Thus, obtained results from AHP, WLC and SMCE susceptibility maps indicated that precipitation was the most important factor in occurrence of landslides in the study area. To confirm the utility of the results, three susceptibility maps were compared with 18 (20%) of 92 landslide zones using R-index and ROC methods. According to the R-index method, when field conditions and attributes are properly described by professional expertise, the SMCE method gave better results and was more accurate than the other two methods in this study. The obtained results identified high percentage for high and very high susceptibility classes in direct association with active landslide zones in the AHP (19.31%), WLC (14.75%), and SMCE (58.49%) maps. Also, the validation results by ROC method show that the area under the curve for AHP, WLC and SMCE models are 0.8719 (87.19%), 0.8417 (84.17%) and 0.9375 (93.75%). From the statistical methods that were used, precision of the method (P) was 96% for SMCE, 91% for FR and 89% for WLC. Thus, the map extracted from the SMCE model is more accurate compared to maps extracted from the other two models.

Generally, the result of validation implies that 86% of the total landslide pixels were properly categorised by the three landslide susceptibility mapping models, which indicates a significant rational carrying out with regard to comparable studies accomplished by other researchers in tropical environments using statistical models. Also, the validation results demonstrated acceptable agreement relating to susceptibility maps and the current data from landslide areas. Furthermore, current research revealed that the C-, L- and P-band images of AIRSAR data are able to provide acceptable coherence over highly vegetated areas. Also, the integration of AIRSAR data with high resolution satellite images can play important role in the production of a landslide inventory map in tropical regions. These landslide susceptibility maps can be used for optimum management by decision makers and land use planners and engineers to decrease losses caused by current and also future landslides through suitable prophylactic assessments and minimization procedures.

## Author Contributions

H.S. wrote the methods and interpretation of results. M.H. contributed to the writing and provided the fund opportunities. All the authors contributed to the interpretation and reviewed the manuscript.

## Figures and Tables

**Table 1:** Pair-wise comparison 9-point rating scale^48^

Importance	Definition	Explanation
1	Equal importance	Contribution to objective is equal
3	Moderate importance	Attribute is slightly favored over another
5	Strong importance	Attribute is strongly favored over another
7	Very strong importance	Attribute is very strongly favored over another
9	Extreme importance	Evidence favoring one attribute is of the highest possible order of affirmation
2, 4, 6, 8	Intermediate values	When compromise is needed

**Table 2:** The class weight, factors weights and consistency ratio of the data layers used by using AHP

Factor	Class	Class weight	Factor weight	Factor	Class	Class weight	Factor weight
Slope (°)	0–10	0.032	0.109	Land cover	Grass	0.081	0.087
	10–20	0.048			Primary forest	0.021	
	20–30	0.084			Rubber	0.132	
	30–40	0.151			Cutting	0.501	
	>40	0.269			Secondary forest	0.348	
					Settlements	0.214	
					Agriculture area	0.120	
	Consistency ratio: 0.043				Water body	0.141	
					Consistency ratio: 0.057		
Aspect	Flat	0.012	0.081	Precipitation (mm)	2612 ~ 2661	0.012	0.142
	North	0.030			2662 ~ 1681	0.018	
	Northeast	0.213			2679 ~ 2694	0.147	
	East	0.201			2695 ~ 2708	0.098	
	Southeast	0.207			2709 ~ 2719	0.847	
	South	0.118			2720 ~ 2731	0.214	
	Southwest	0.123			2732 ~ 2743	0.425	
	West	0.173			2744 ~ 2754	0.317	
	Northwest	0.024			2755 ~ 2764	0.306	
	Consistency ratio: 0.061				2765 ~ 2781	0.624	
					Consistency ratio: 0.039		
Soil	Serong series	0.325	0.093	Distance to road (m)	0–50	0.435	0.078
					50–100	0.321	
	Alluvium-colluvium	0.127			100–200	0.145	
					200–500	0.101	
	Consistency ratio: 0.078				>500	0.089	
					Consistency ratio: 0.055		
Lithology	Metamorphic rock	0.498	0.124	Distance to drainage (m)	0–50	0.341	0.089
					50–100	0.234	
	Igneous rock	0.102			100–150	0.217	
					150–200	0.127	
					200–300	0.084	
					300–500	0.051	
	Consistency ratio: 0.071				>500	0.030	
					Consistency ratio: 0.063		
NDVI	−0.774 ∼ –0.613	0.302	0.101	Distance to fault (m)	0–50	0.214	0.074
	−0.618 ∼ –0.459	0.274			50–100	0.178	
	−0.457 ∼ –0.303	0.151			100–150	0.098	
	−0.309 ∼ –0.139	0.092			150–200	0.052	
					200–500	0.049	
	−0.144 ~ 0.012	0.067			>500	0.021	
	0.015 ~ 0.174	0.049					
	0.172 ~ 0.328	0.032					
	0.332 ~ 0.491	0.021					
	0.491 ~ 0.648	0.051					
	0.641 ~ 0.809	0.071					
	Consistency ratio: 0.066				Consistency ratio: 0.049		

**Table 3:** Pair-wise comparison matrix, factor weights and consistency ratio of the data layers

Factors	Slope	Aspect	Soil	Lithology	NDVI	Land cover	Precipitation	Distance to road	Distance to drainage	Distance to fault	Weights
Slope	1										0.123
Aspect	1	1									0.102
Soil	5	4	1								0.121
Lithology	2	1/2	1/5	1							0.097
NDVI	5	5	2	3	1						0.073
Land cover	3	5	1/2	4	1/2	1					0.086
Precipitation	2	3	1/5	3	1/2	1/3	1				0.141
Distance to road	5	6	2	5	3	3	5	1			0.084
Distance to drainage	4	4	1/3	3	1/4	1/3	3	1/5	1		0.081
Distance to fault	3	3	1/5	2	1/5	1/4	2	1/5	1/2	1	0.062
Consistency ratio: 0.069 < 0.1 (acceptable)											

**Table 4:** The weight value of each group and weight value of factors using pairwise comparison for the SMCE model

Group factor	Weights value of group	Factor	Weights value of factor	Fuzzy membership	Standardized method
Geomorphological	0.132	Slope	0.128	0.92	Concave
		Aspect	0.112	0.94	Interval
Geological	0.871	Distance to fault	0.073	0.98	Maximum
		Lithology	0.097	0.92	Interval
Environmental	0.124	Soil	0.123	0.85	Concave
		Land cover	0.084	0.95	Interval
		Distance to road	0.083	1.00	Maximum
		NDVI	0.087	0.83	Maximum
Hydrological	0.146	Distance to drainage	0.081	0.81	Maximum
		Precipitation	0.143	0.79	Maximum
Consistency ratio	0.057	Consistency ratio	0.061		

**Table 5:** Comparison of the information obtained from crossing each of the susceptibility maps with the map of landslides distribution

Method of slide susceptibility map	susceptibility classes	Si (Km^2^)	Ai (Km^2^)	Density of slide in any class	Density of slide in the whole map	Σ*_l_n* (Si/Ai)	Landslide index (Li) in any class percent	Ks (km^2^)	S (km^2^)	P
AHP	Low	1.57	9.27	0.10	0.20	0.100	28.32	1.62	6.27	0.84
	Moderate	3.08	19.09	0.24			36.23			
	High	1.09	8.33	0.12			24.18			
	Very high	0.53	1.71	0.08			11.27			
WLC	Low	1.17	8.12	0.11	0.20	0.100	22.34	1.07	6.27	0.79
	Moderate	4.03	24.33	0.45			46.56			
	High	0.89	4.94	0.08			17.12			
	Very high	0.18	1.01	0.01			7.10			
SMCE	Low	0.41	1.10	0.05	0.20	0.100	10.16	5.25	6.27	0.96
	Moderate	0.61	2.87	0.09			13.18			
	High	1.93	11.34	0.13			29.14			
	Very high	3.32	23.09	0.39			47.52			
